# *Steinernema poinari* (Nematoda: Steinernematidae): a new symbiotic host of entomopathogenic bacteria *Xenorhabdus bovienii*

**DOI:** 10.1007/s00203-018-1544-9

**Published:** 2018-06-26

**Authors:** Ewa Sajnaga, Waldemar Kazimierczak, Marcin Skowronek, Magdalena Lis, Tomasz Skrzypek, Adam Waśko

**Affiliations:** 1 Laboratory of Biocontrol, Application and Production of EPN, Faculty of Biotechnology and Environmental Sciences, Centre for Interdisciplinary Research, John Paul II Catholic University of Lublin, Konstantynów 1J, 20-708 Lublin, Poland; 2 Laboratory of Confocal and Electron Microscopy, Faculty of Biotechnology and Environmental Sciences, Centre for Interdisciplinary Research, John Paul II Catholic University of Lublin, Konstantynów 1J, 20-708 Lublin, Poland; 3 Department of Biotechnology, Microbiology and Human Nutrition, University of Life Sciences in Lublin, Skromna 8, 20-704 Lublin, Poland

**Keywords:** *Xenorhabdus*, *Steinernema*, Entomopathogenic nematodes, Symbiosis, Symbiont exchange

## Abstract

Three strains of symbiotic bacteria were isolated from an entomopathogenic nematode *Steinernema poinari* retrieved from soil in eastern Poland. Using 16S rDNA, *recA, gltX, gyrB*, and *dnaN* gene sequences for phylogenetic analysis, these strains were shown to belong to the species *Xenorhabdus bovienii*. The nucleotide identity between the studied *S. poinari* microsymbionts and other *X. bovienii* strains calculated for 16S rDNA and concatenated sequences of four protein-coding genes was 98.7–100% and 97.9–99.5%, respectively. The phenotypic properties of the isolates also supported their close phylogenetic relationship with *X. bovienii*. All three tested *X. bovienii* strains of different *Steinernema* clade origin supported the recovery of infective juveniles and subsequent development of the nematode population. However, the colonization degree of new infective juvenile generations was significantly affected by the bacterial host donor/recipient. The colonization degree of infective juveniles reared on bacterial symbionts deriving from a non-cognate clade of nematodes was extremely low, but proved the possible host-switching between non-related *Steinernema* species.

**Electronic supplementary material:**

The online version of this article (10.1007/s00203-018-1544-9) contains supplementary material, which is available to authorized users.

## Introduction

Bacteria of the genus *Xenorhabdus* in symbiotic association with entomopathogenic nematodes (EPN) of the genus *Steinernema* Travassos (Nematoda: *Steinermatidae*) infect a wide range of soil-dwelling insects (Koppenhöfer and Gaugler 2009; Stock 2015). They have an unusual property of switching from a mutualistic to pathogenic lifestyle interacting with two different eukaryotic hosts and this triplicate (bacteria–nematode–insect) system can be easily established and investigated in the laboratory conditions. Bacteria of the genus *Xenorhabdus* are isolated from the intestinal lumen of their nematode host or infected insects as their natural habitats; they have never been found in free-living stage in the soil. Isolation and classification of EPN symbiotic bacteria were performed for the first time in 1964 and 26 species of the genus *Xenorhabdu*s have been described to date (Table S1, supplementary material).

EPN and their microsymbionts have become a successful biological model facilitating investigation of prokaryote–eukaryote interactions (especially virulence mechanisms and a variety of bacterial natural products) as well as soil ecology (Campos-Herrera et al. 2012; Goodrich-Blair and Clarke 2007; Stock 2005). Additionally, both EPN and their bacterial symbionts play an important role in crop protection against insect pests, and identification of native nematode–bacteria associations is essential for successful control of pests in a particular area (Hiltpold 2015). Previously reported results indicated that most *Xenorhabdus* species are highly pathogenic to insects when directly injected without their nematode host (e.g. McMullan et al. 2017a; Sicard et al. 2004a, b; Sugar et al. 2012). This enhances the use of the bacteria (or only their genes) producing insecticidal toxins for biological control in agroforestry industries (Kumari et al. 2015; McMullen et al. 2017a; Zhang et al. 2012). Strains of some genus *Xenorhabdus* species can display a broad spectrum of genetic diversity. They differ in the virulence phenotype, which is determined by a wide variety of secreted bioactive components necessary for invasion, colonization, and use of the insect cadaver as a food source. Analyses of different *Xenorhabdus* strains increase the potential for discovery of novel natural products, including antibiotics, toxins, adhesins, hemolysins, proteases, and lipases (Hinchliffe et al. 2010; Murphin et al. 2015a). The increasing number of genus *Xenorhabdus* strains that are being sequenced is also a key factor in exploration thereof in medicine and pharmacy.

Although *Xenorhabdus* strains have the potential to become commercially important, the knowledge of the bacteria of the genus *Xenorhabdus*, especially their diversity within the species, is still insufficient. Many symbiotic bacteria of EPN have not been identified yet, which obscures comprehensive characterization of these organisms and limits their application.

The symbiotic *Xenorhabdus*–*Steinernema* association is specific, and each EPN species associates with only one *Xenorhabdus* species. However, most *Xenorhabdus* species display more flexibility in the nematode symbiont choice and may be associated with more than one *Steinernema* species (Stock 2015; Table S1, supplementary material). Previous studies have shown that *X. bovienii* bacteria can enter symbiosis with 14 EPN species, which makes such a broad host range a highly significant feature of this species. The EPN hosts of *X. bovienii* are distributed widely all over the world. They belong to two distinct clades of *Steinernema* distinguished by Nadler et al. (2006) on the basis of the sequences of nuclear and mitochondrial genes: *affine*–*intermedium* (clade I) and *feltiae–kraussei* (clade III). These nematodes constitute about half of the Steinernema species described so far.

*Steinernema poinari* was found for the first time in the Czech Republic, but it is quite abundant in Europe, also in Poland. Molecular and morphological characteristics have shown that this species is a member of the *affine–intermedium* group (Mráček et al. 2014). The genus *Xenorhabdus* bacteria described in this paper were isolated from three strains of *S. poinari* recovered in Poland. To reveal the taxonomic position of *S. poinari* microsymbionts, we analysed five loci 16S rDNA, *recA, gltX, gyrB*, and *dnaN*. The bacteria were also characterized phenotypically using biochemical and physiological tests. To date, there are no reports on *S. poinari* bacterial symbionts and this study is the first to provide information on their identification, phylogenetic relatedness, and phenotypic characteristics. We also performed experiments on the influence of different origin *X. bovienii* strains on EPN to uncover the possibility of symbiont exchange in this beneficial partnership.

## Materials and methods

### Bacterial isolation and phenotypic tests

The *Xenorhabdus* spp. strains used in this study were symbiotically associated with Polish isolates of *S. poinari* (Nematoda: *Steinernematidae*) (Table S2, supplementary material). All nematode strains were isolated from soil samples using the live trap method (Akhurst and Bedding 1975). Before isolation of the symbiotic bacteria, straight genetic lines of the nematodes were established [offspring of two infective juveniles (IJs)] and identified (microscopically and molecularly). The bacterial strains were isolated from the hemolymph of *Galleria mellonella* larvae (Lepidoptera: Pyralidae) as previously described (Kazimierczak et al. 2016). They were designated Xb041, Xb057, and Xb139 and are maintained in our laboratory collection. All biochemical and physiological tests were performed twice at 25 °C according to Kazimierczak et al. (2016).

### Gene sequencing

Bacterial genomic DNA was extracted using a Genomic Mini AX Bacteria Spin Kit (A&A Biotechnology). 16S rDNA was amplified using primers 16SP1 and 16SP2 (Tailliez et al. 2006). The housekeeping genes coding the glutamyl-tRNA synthetase catalytic subunit (*gltX*), recombinase A (*recA*), DNA polymerase III beta chain (*dnaN*), and subunit B of DNA gyrase (*gyrB*) were amplified as described earlier (Kazimierczak et al. 2016). The PCR products were ligated into plasmids pJET1.2 (Thermo Fisher Scientific) according to the manufacturer’s instructions. The plasmids were transformed in *E. coli* XL1 Blue using a standard method. The inserts of positive clones were sequenced from both strands in Genomed (Poland).

### Phylogenetic analysis

The 16S rRNA, *gltX, recA, dnaN*, and *gyrB* gene sequences of isolates Xb041, Xb057, and Xb139 obtained in this study were compared to GenBank nucleotide sequences using BLAST available on the NCBI website. Multiple sequence alignments were created using ClustalW at the default configuration. The evolutionary history of the studied *Xenorhabdus* strains was inferred using the neighbor-joining method in MEGA 6.06 (Tamura et al. 2004, 2013). The evolutionary distances were computed using the Tamura-Nei algorithm. All positions containing gaps and missing data were eliminated. To determine the statistical support for branches, bootstrapping with 1000 replicates of the data was conducted. There were 1334 positions in the final dataset for 16S rDNA, 382 for *recA*, 783 for *dnaN*, 1006 for *gltX*, and 811 for *gyrB*. The GeneBank accession numbers for the gene sequences specified in this study are as follows: MG995576-78 for 16S rDNA, MH001597-99 for *recA*, MH001600-02 for *gyrB*, MH001594-96 for *dnaN*, and MH001603-05 for *gltX* (Table S2, supplementary material).

### Bacterial/EPN host interaction bioassays

We performed experiments on the influence of different origin symbiotic bacteria on (i) the recovery of IJs (termination of developmental diapause), (ii) nematode population development, and (iii) colonization degree of IJs (percent of IJs possessing a bacterial symbiont in their intestines). In these experiments, we used single bacterial strains symbiotically associated with *S. poinari* (strain Xb057), *S. affine* (strain Xb-aff), *S. intermedium* (strain Xb-int) (the same nematode clade), *S. feltiae* (strain Xb-fel), and *S. silvaticum* (strain Xb-Z1Z) (another *Steinernema* clade symbiotically associated with *X. bovienii*). The nematodes were reared on Petri dishes with modified Wouts agar (1981) [1% (w/v) full-fat soy flour, 1% (w/v) lyophilized yeast, 1% (w/v) dried egg yolk, 2% (w/v) agar, 0.5% (w/v) NaCl, 1000 ml deionized water]. After 48 h of bacterial growth at 25 °C, the plates were inoculated with ~ 100 of surface-sterilized IJs [three 15-min washes in a 0.4% (w/v) Hyamine® solution, two 5-min washes in sterile deionized water]. The plates with the nematodes were placed on modified White traps (Stock and Goodrich-Blair 2012) at 17.5 °C and were regularly checked. After 2–3 weeks, new generations of nematodes were harvested from White traps and stored in tap water at 12 °C. The colonization degree of IJs was determined for groups of 20 IJs and insects separately, dividing the number of dead insects by the number of infected insects (dead and live) as previously described (Kazimierczak et al. 2018). For each combination of the bacteria and nematode, the colonization degree was determined for five groups of IJs and insects. All experiments were repeated twice with the use of nematodes and *G. mellonella* larvae originating from different batches.

### Statistical analysis

Since there was no effect of the experiment replication on the nematode colonization degree, the data from the two replications of the experiment were combined for the analysis. Before analysis, the data were normalized by arcsin square root transformation. The normality of data distribution was determined with the Shapiro–Wilk test and homogeneity of variance was assessed with Levene’s test. Analysis of variance and means separation with Tukey HSD test were used for comparisons. Back-transformed means ± SE are presented. All analyses were conducted using SPSS Statistics 24 software (IBM).

## Results and discussion

### Phylogeny of the *S. poinari* microsymbionts based on 16S rDNA

**Fig. 1:**
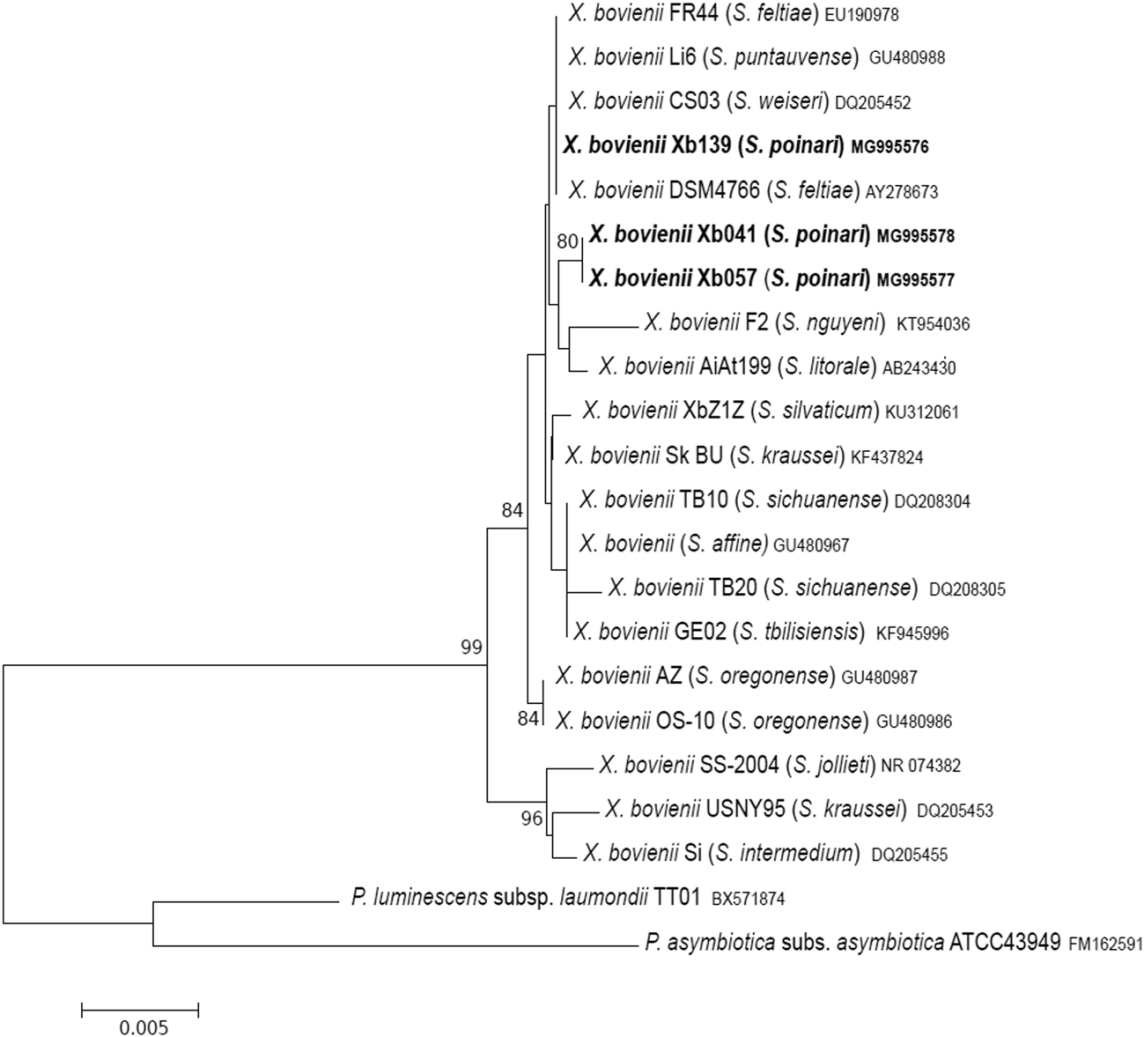
Neighbor-joining tree showing the phylogenetic relationships of the *S. poinari* microsymbionts studied (bolded) with *X. bovienii* strains based on 16S rRNA gene sequences. Bootstrap values based on 1000 replicates > 70% are indicated at the branching points. The scale bar presents the number of nucleotide substitutions per site. The sequences of *Photorhabdus luminescens* subsp. *laumondii* and *Photorhabdus asymbiotica* subsp. *asymbiotica* were used as an outgroup

The use of the 16S rRNA gene has proved useful for classification, identification, and characterization of entomopathogenic nematode bacteria. Nevertheless, many studies have shown such confounding factors in 16S rRNA phylogeny as evidence of lateral gene transfer (LTG) and a low level of variation between 16S rDNA sequences, including *Xenorhabdus* spp. (Lee and Stock 2010a; Tailliez et al. 2006). It is generally accepted that 16S rRNA gene sequence similarity of two strains < 98.7 indicates that they belong to different species, but there is no defined threshold of 16S rDNA similarity above which it is possible to identify bacteria at a species level (Stackebrandt and Ebers 2006; Yarza et al. 2014). The sequences of 16S rDNA obtained for Xb041, Xb057, and Xb139 isolates shared 99.8–100% sequence similarity with each other (maximum 3 bp differences), which placed all the isolates in the same species. The analysed sequences were most similar to the 16S rDNA sequences reported for *X. bovienii* displaying 98.7–100% identity with them (max. 16 nucleotide substitutions). The phylogenetic analysis of the 16S rDNA sequences divided the *X. bovienii* strains and our isolates into two groups. All of the Polish S. *poinari* isolates were placed in a large cluster together with X. *bovienii* strains being symbiotic partners of nematodes from both groups *feltiae–kraussei* and *affine*–*intermedium* (Fig. 1).

### Phylogeny of the *X. bovienii* isolates based on the combination of *recA, gltX, gyrB*, and *dnaN* gene fragments

**Fig. 2:**
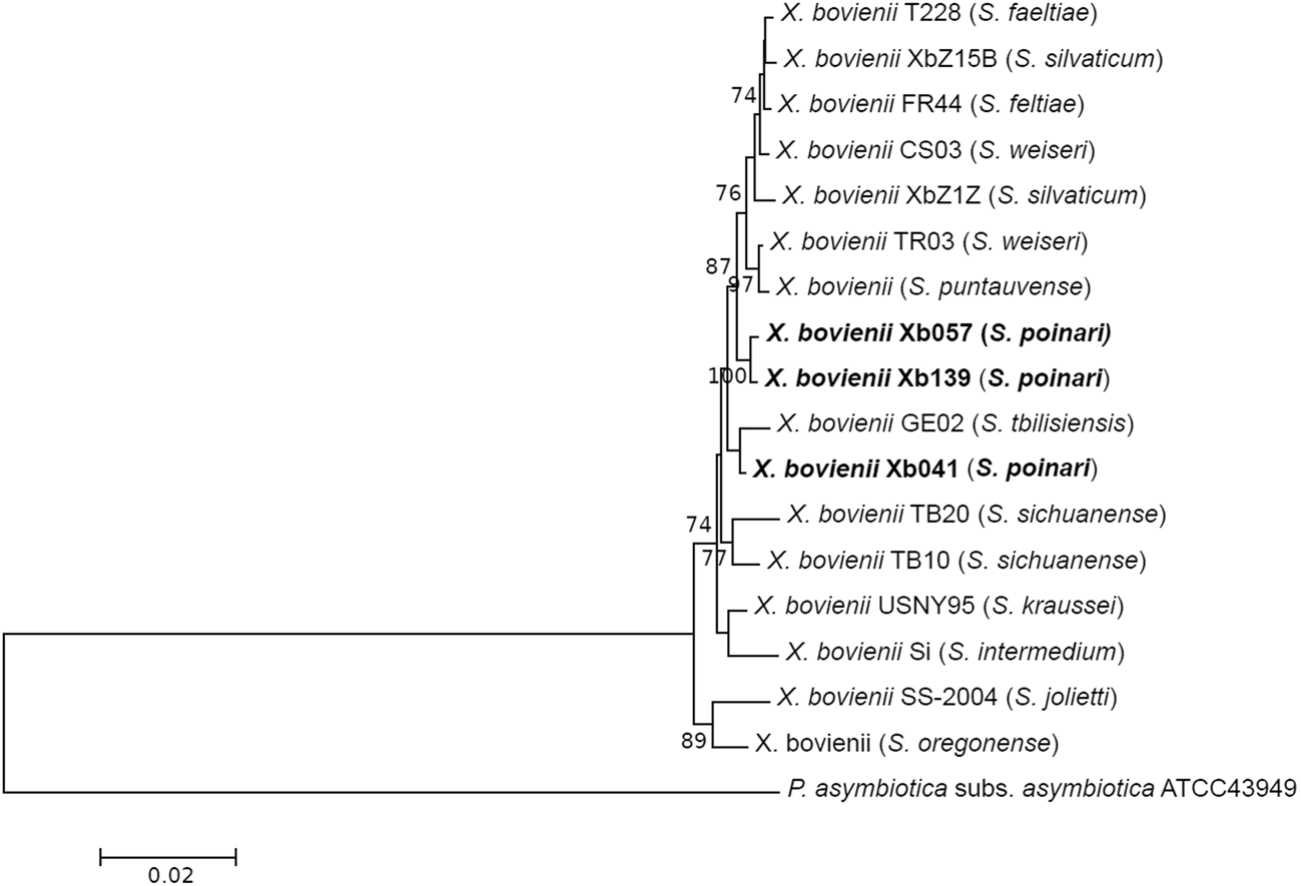
Neighbor-joining tree showing the phylogenetic relationships of *S. poinari* microsymbionts studied (bolded) with *X. bovienii* strains based on concatenated *recA, gyrB, dnaN*, and *gltX* gene sequences. Bootstrap values based on 1000 replicates > 70% are indicated at the branching points. The scale bar presents the number of nucleotide substitutions per site. The sequence of *Photorhabdus asymbiotica* subsp. *asymbiotica* was used as an outgroup

Taking into account the conservative nature of 16S rDNA sequences between *X. bovienii* strains, additional phylogenetic information was derived from the analysis of four housekeeping gene sequences. This multi-gene approach increases the discriminatory power of phylogenetic analysis and prevents misclassification of new isolates. In particular, comparative sequence analysis of *recA, gltX, gyrB*, and *dnaN* has been widely used for the diagnostics of entomopathogenic bacteria species (e.g. Dreyer et al. 2017; Ferreira et al. 2013; Tailliez et al. 2012). On the basis of grouping obtained with *recA, gyrB, dnaN*, and *gltX* gene sequences, Tailliez et al. (2010) have demonstrated that all *X. bovienii* strains constitute a separate clade within the genus *Xenorhabdus*. This broad phylogenetic study has provided evidence that the genus *Xenorhabdus* strains that shared less than 97% nucleotide identity of the concatenated sequences of the *recA, gyrB, dnaN* and *gltX* do not belong to the same species. In our analysis, concatenated 2990-bp-long sequences of these genes for the Polish isolates of *S. poinari* shared 99.2–99.8% similarity with each other and 97.9–99.5 with those reported for *X. bovienii*. In the phylogram constructed on the basis of the concatenated sequences of *recA, gltX, gyrB*, and *dnaN* genes (Fig. 2), all the Polish *S. poinari* isolates formed a homogeneous and highly supported cluster with other *X. bovienii* strains. This confirms their identification as *X. bovienii*.

It is known that the sequences of *recA, gltX, gyrB*, and *dnaN* genes within the genus *Xenorhabdus* are highly conserved and display similarity > 80% (Tailliez et al. 2010). The weak phylogenetic signal of each individual housekeeping gene usually resulted in low bootstrap values and a high incidence of unresolved branches in the evolutionary analysis of *Xenorhabdus* (Lee and Stock 2010a). To assess the amount of phylogenetic information associated with each protein-coding gene analysed, we determined the number of variable and parsimony-informative sites in single-gene alignments. Gene *gltX* had the highest number of parsimony-informative positions among the analysed *X. bovienii* strains − 44 (4.4%), and the lowest number was recorded for *recA* − 7 positions (1.8%) and *gyrB* − 13 (1.6%). The studied Polish *S. poinari* microsymbionts showed a high level of *gyrB, dnaA*, and *recA* gene conservation—maximum double and single synonymous substitution was detected for *gyrB* and *dnaN*, respectively, whereas the *recA* gene sequences were identical. In contrast, the *gltX* gene in the studied isolates was more divergent—strain Xb041 differed from Xb057 and Xb139 by 17 and 14 positions, respectively. Interestingly, Xb041 displayed 100% similarity in the *gltX* gene sequence to *X. bovienii* GE02, a bacterial symbiont of *S. tbilisiensis*. This resulted in grouping together of the Xb041 isolate and the *X. bovienii* GE02 strain on the *gltX* tree in a distant position from the other bacteria included in the analysis (Fig. S1). This relationship was not revealed by the phylogenetic analysis of the 16S rDNA, *recA*, and *dnaN*; according to the sequences of these loci, our isolates form a homogenous group with the *X. bovienii* strains (Fig. 1, Fig. S3, Fig. S4). In turn, the *gyrB* gene of the studied isolates displayed relatively high similarity to the *X. bovienii* GE02 *gyrB* gene (1–3 substitutions), resulting in their placement in the same clade on the phylogram (Fig. S2). The discrepancies in individual gene trees may be a result of LTG or different evolutionary pressures on the gene studied. Despite the high resistance of housekeeping genes to LTG, the occurrence of recombination for *gltX, gyrB*, and *serC* within the *Xenorhabdus* clade is known (Sergeant et al. 2006; Tailliez et al. 2010). It supports the assumption that the ambiguous phylogenetic relationships among the studied strains could be caused by lateral gene transfer.

### Phenotypic characterization of bacterial isolates

All the tested strains of the genus *Xenorhabdus* were Gram-negative, catalase-, urease- and cytochrome oxidase-negative rods. The bacterial strains used in our study absorbed bromothymol blue from NBTA and neutral red from MacConkey agar plates. The *Xenorhabdus* colonies growing on the LBA plates were yellow and the size of bacterial cells was variable (2.4–8.3 µm). The maximum growth temperature on the LBA plates was in the range of 32–33 °C. The tested bacterial strains were motile, ampicillin resistant, and able to β-haemolyse on sheep blood agar and hydrolyse casein and gelatin. The *Xenorhabdus* isolates associated with *S. poinari* were arginine dihydrolase, DNase, and phospholipase positive. All the strains metabolized *N*-acetyl-d-glucosamine, α-d-glucose, d-mannose, d-trehalose, d-gluconic acid, inosine, uridine, glucose-1-phosphate, and glucose-6-phosphate in aerobic conditions. Tween®80, methyl pyruvate, p-hydroxy-phenylacetic acid, d,l-lactic acid, l-alanine, l-alanyl-glycine, l-asparagine, l-histidine, glycerol and d,l-α-glycerol phosphate were utilized weakly. The bacterial strains used in this study fermented d-glucose, d-mannose, and *N*-acetylglucosamine. d-Trehalose, potassium gluconate, and potassium-5-ketogluconate were fermented weakly. The results of the other oxidation and fermentation tests were strain dependent (compare Table 1). All the tested strains of the genus *Xenorhabdus* exhibited phenotypic characteristics typical of *X. bovienii* bacteria (Boemare and Akhurst 1988; Tailliez et al. 2006), which confirms their molecular identification.

**Table 1:** Phenotypic characteristics of symbiotic bacteria isolated from Polish strains of *S. poinari*

General characterization									
	Bacterial strain				Bacterial strain				
	Xb041	Xb057	Xb139		Xb041	Xb057			Xb139
Gram staining	−	−	−	Pigmentation	Yellow	Yellow			Yellow
Bromothymol blue from NBTA	+	+	+	Neutral red from MacConkey agar	+	+			+
Cell length (µm)	4.6 ± 1.3 [2.5/8.3]	4.2 ± 1.0 [2.4/7.1]	4.7 ± 1.4 [3.3/8.0]	Cell width (µm)	1.2 ± 0.2 [0.9/1.6]	1.5 ± 0.2 [1.1/1.8]			1.7/0.2 [0.9/1.8]
Maximum temperature for growth (LB) (°C)	32	32	33	Motility	+	+			+
Ampicillin resistance	+	+	+	Hemolysis type	β	β			β
Arginine dihydrolase	+	+	+	Catalase	−	−			−
Cytochrome oxidase	−	−	−	DNase	+	+			+
Phospholipase	−	−	−	Urease	−	−			−
Proteolysis (casein)	+	+	+	Proteolysis (gelatin)	+	+			+
Oxidation/fermentation (GN2)									
Carbon source				Carbon source					
Tween 80	w	w	w	l-Asparagine	w	w			w
*N*-Acetyl-d-galactosamine	w	+	+	l-Aspartic acid	w	−			w
*N*-Acetyl-d-glucosamine	+	+	+	l-Glutamic acid	+	+			w
d-Fructose	+	w	w	l-Histidine	w	w			w
α-d-Glucose	+	+	+	l-Proline	w	−			−
d-Mannose	+	+	+	d-Serine	−	w			−
d-Trehalose	+	+	+	l-Serine	−	w			−
Methyl pyruvate	w	w	w	Inosine	+	+			+
d-Gluconic acid	+	+	+	Uridine	+	+			+
*P*-Hydroxy-phenylacetic acid	w	w	w	Putrescine	w	−			w
d,l-Lactic acid	w	w	w	Glycerol	w	w			w
l-Alaninamide	w	−	w	d,l-α-Glycerol phosphate	w	w			w
d-Alanine	w	−	w	Glucose-1-phosphate	+	+			+
l-Alanine	w	w	w	Glucose-6-phosphate	+	+			+
l-Alanyl-glycine	w	w	w						
Fermentation (API50CH)									
Carbon source				Carbon source					
Glycerol	w	+	+	*N*-Acetylglucosamine	+		+	+	
d-Ribose	−	−	+	d-Maltose	+		w	+	
d-Glucose	+	+	+	d-Trehalose	w		w	w	
d-Fructose	w	+	+	Potassium gluconate	w		w	w	
d-Mannose	+	+	+	Potassium 5-ketogluconate	w		w	w	
Inositol	−	−	w						

*+* positive, *w* week, *−* negative

### Influence of different *X. bovienii* strains on the entomopathogenic nematodes

**Fig. 3:**
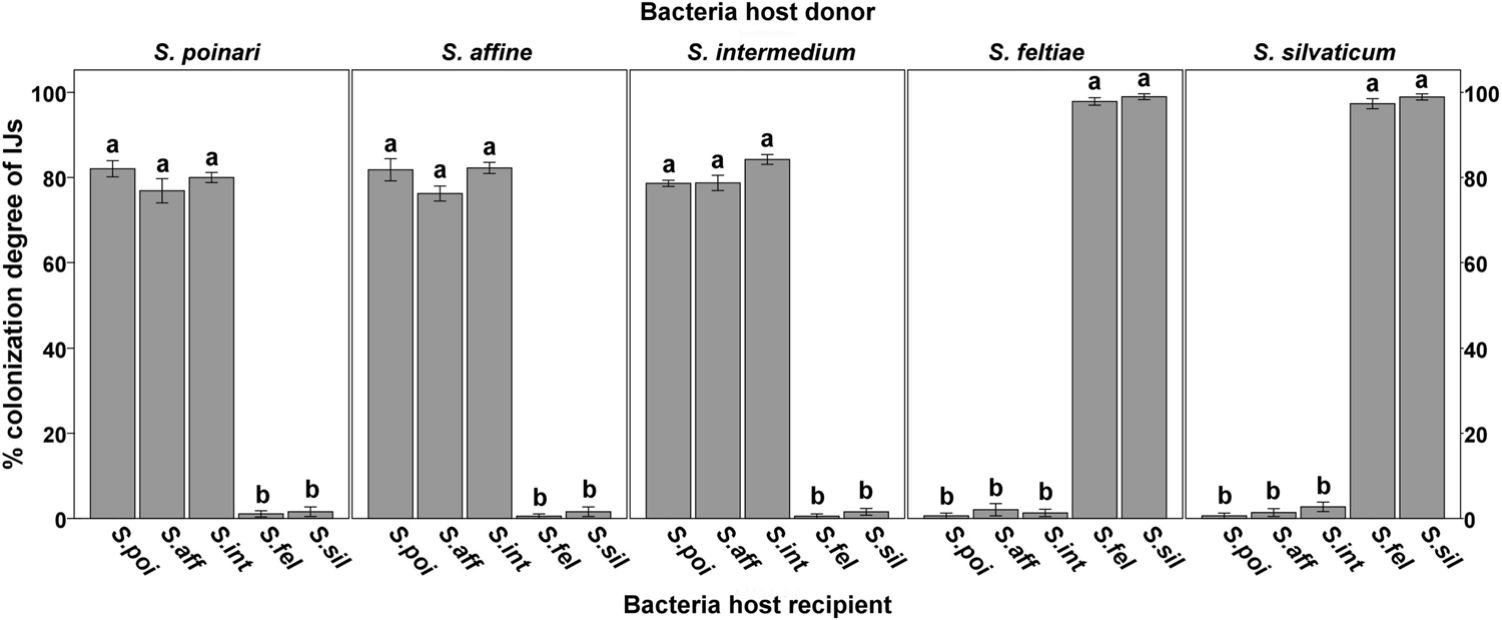
Effect of native- and non-native bacteria on the mean (± SE) colonization degree of *Steinernema* spp. IJs. Columns with the same letter are not significantly different within the bacterial host donor (*P* < 0.05, Tukey HSD test). *S. aff*—*S. affine, S. fel*—*S. feltiae, S. int*—*S. intermedium, S. poi*—*S. poinari, S. sil*—*S. silvaticum*

All the tested strains of *X. bovienii* supported the recovery of IJs and subsequent development of the nematode population. However, the colonization degree of the new IJ generations was significantly affected by the bacterial host donor/recipient (*F*_4,45_≥617.76, *P* < 0.001, Fig. 3). There was no significant effect of the *X. bovienii* strains on the colonization degree of harvested IJs when the bacterial donor and recipient were derived from the same clade of nematodes. In these cases, the colonization degree of IJs was ~ 80% for the *affine–intermedium* clade and > 95% for the *feltiae–kraussei* clade. When the nematodes were reared on bacteria from another clade of *Steinernema*, the mean colonization degree of harvested IJs was low (0.5/2.7%).

Phylogenetic data demonstrate that strains of *X. bovienii* are often grouped together on phylogenetic trees regardless of the phylogenetic origin of their nematode host. For example, the *X. bovienii* Si and SS-2004 strains are derived from phylogenetically distant hosts (S. *intermedium* from clade I and *S. jollieti* from clade III, respectively), but they occupy the same place in the phylogenetic trees. A similar trend has been noted in the tested Xb041, Xb057, and Xb139 microsymbionts of *S. poinari* from clade I—they are phylogenetically very close to the strains of *X. bovienii* isolated from *Steinernema* spp. belonging to clade III (Figs. 1, 2). These results suggest a low level of specificity in the symbiotic interaction of *X. bovienii* and their *Steinernema* partners and strengthen the hypothesis of strong host-switching shaping these relationships. This is in agreement with the previous observation of host switches in the case of *X. bovienii*, a symbiont of *S. affine* and *S. intermedium* (Lee and Stock 2010b). The ability to switch between nematodes from distantly related clades has also been reported for *X. khoisanae* or *X. nematophila* bacteria (Dreyer et al. 2017; Stock 2015). However, a cophylogenetic study of 30 *Steinernema*/*Xenorhabdus* pairs revealed 17 apparent mismatches between two trees, indicating host-switching as well as 12 cospeciation events. This suggests that a high level of specificity between the nematode host and the bacterial partner can occur for some host–symbiont phylogenetic relationships (Lee and Stock 2010b). Similarly, another cophylogenetic analysis has indicated specificity and coevolution in symbiotic partner pairs as evidenced by remarkable congruence between the phylogenies of nematodes from the *bicornutum* group of *Steinernema* and their symbiotic bacteria (Bhat et al. 2018) as well as between *S. feltiae* and *S. puntauvense*/*X. bovienii* associations (Murfin et al. 2015b). A high level of specificity with limited horizontal transfers between these symbiotic partners was also suggested in a study conducted by Emelianoff et al. (2008), which showed limited transfers of *X. bovienii* bacteria between *S. feltiae* and *S. affine* living in the same area.

The results of the switches of *Xenorhabdus* between different *Steinernema* species in laboratory experiments also vary depending on the pairs considered. However, the experimental tests revealed that closely related bacterial strains provide nematodes with greater virulence than more phylogenetically divergent ones. The reproductive fitness of both symbionts decreased as the phylogenetic distance between native and non-native nematode partner increased (McMullen et al. 2017b; Murfin et al. 2015b).

Symbiosis of numerous nematode species with *X. bovienii* can reduce interspecific competition between *Steinernema* species, increasing the size of potential food resources. It can also explain the worldwide occurrence and frequent coexistence of *affine–intermedium* and *feltiae–kraussei Steinernema* clades (Půža and Mráček 2010) and probably frequent host-switching events (Murfin et al. 2015b). However, the results of our study indicate that when the donors and recipients of *X. bovienii* represent a different nematode clade, the colonization degree of newly formed IJs is very low. This proves that host-switching events are rare, but possible. Previously published studies (Grewal et al 1997; Sicard et al. 2004a, b) have shown that secondary infection of insects by nematodes from another *Steinernema* clade leads to termination of nematode diapause, their population development, and formation of new IJs. These new IJs, as we have shown, have much lower competitive abilities because most of them are axenic. Axenic IJs are able to infect a new host, but without symbiotic bacteria they are not able to reproduce and fully utilize food resources. Host co-infection or secondary infection by nematodes from different *Steinernema* clades symbiotically associated with *X. bovienii* may, therefore, lead to reduction of their competitive abilities. The mechanisms of the coexistence of competitor populations remain unknown and will be investigated.

## Conclusions

We found that entomopathogenic nematodes *S. poinari* are symbiotically associated with *X. bovienii*. This result increases the known range of *X. bovienii* hosts and confirms that nematodes from the *affine–intermedium* and *feltiae–kraussei* clades of *Steinernema* enter symbiosis with *X. bovienii* exclusively. All three strains of *X. bovienii* used in our experiments supported the recovery of IJs and development of the nematode population. The colonization degree of IJs reared on bacterial symbionts deriving from a non-cognate clade of nematodes was extremely low, but proved the possible host-switching of *X. bovienii* between non-related *Steinernema* species.

## Electronic supplementary material

Below is the link to the electronic supplementary material.

Supplementary material 1 (PDF 856 KB)

## Abbreviations

IJ (s): Infective juvenile (s)
EPN: Entomopathogenic nematode (s)
BLAST: Basic Local Alignment Search Tool
NCBI: National Center for Biotechnology Information
w/v: Weight per volume
NBTA: Nutrient bromothymol blue agar
LBA: Luria–Bertani agar
SE: Standard error
LTG: Lateral gene transfer
