# Supplementary material for: Steinernema poinari (Nematoda: Steinernematidae): a new symbiotic host of entomopathogenic bacteria Xenorhabdus bovienii
Source: Arch Microbiol. 2018 Jun 26;200(9):1307–16. doi: 10.1007/s00203-018-1544-9 (PMC6182613; doi:10.1007/s00203-018-1544-9)
Supplement: Supplementary file 1 — Supplementary material 1 (PDF 856 KB) [file 203_2018_1544_MOESM1_ESM.pdf]

Supplementary material

Title:

Steinernema poinari (Nematoda:Steinermatidae) - a new symbiotic host of entomopathogenic bacteria *Xenorhabdus bovienii*

Journal name:

Archives of Microbiology

Author (s) names:

Ewa Sajnaga[a*], Waldemar Kazimierczak[a] Marcin Skowronek[a], Magdalena Lis[a], Tomasz Skrzypek[b], Adam Waśko[c]

[a] Laboratory of Biocontrol, Application and Production of EPN, Centre for Interdisciplinary Research, Faculty of Biotechnology and Environment Sciences, John Paul II Catholic University of Lublin, Konstantynów 1J, 20-708 Lublin, Poland

[b] Laboratory of Confocal and Electron Microscopy, Centre for Interdisciplinary Research, Faculty of Biotechnology and Environment Sciences, John Paul II Catholic University of Lublin, Konstantynów 1J, 20-708 Lublin, Poland

[c] Department of Biotechnology, Microbiology and Human Nutrition, University of Life Sciences in Lublin, Skromna 8, 20-704 Lublin, Poland

5. Corresponding author:

Ewa Sajnaga, Laboratory of Biocontrol, Application and Production of EPN, Centre for Interdisciplinary Research, Faculty of Biotechnology and Environment Sciences, John Paul II Catholic University of Lublin, Konstantynów 1J, 20-708 Lublin, Polanand; esajnaga@kul.pl, tel. +48 81 454 56 25, fax. +48 454 56 35

Table S1. List of the currently described genus *Xenorhabdus* species and their EPN hosts

***Xenorhabdus* species**	**References**	***Steinernema* species being host**
X. beddingii	Akhurst and Boemare 1988	Two undescribed species
X. bovienii	Akhurst and Boemare 1988	*S. feltiae, S. kraussei, S. affine, S. intermedium, S. weiseri, S. silvaticum, S. sichuanense, S. nguyeni, S. tbilisiensis, S. jollieti, S. puntauvense, S. oregonense, S. litorale*
X. budapestensis	Lengyel et al. 2005	*S. bicornutum, S. ceratophorum*
X. cabanillasii	Tailliez et al. 2006	*S. riobrave*
X. doucetiae	Tailliez et al. 2006	*S. diaprepesi*
X. eapokensis	Kämpfer et al. 2017	*S. eapokensis*
X. ehlersii	Lengyel et al. 2005	*S. longicaudum* (*S. serratum**)
X. griffiniae	Tailliez et al. 2006; Dreyer et al. 2017	*S. hermaphroditum,* one undescribed species
X. hominickii	Tailliez et al. 2006	*S. karii, S. monticolum*
X. indica	Somvanshi et al. 2006	*S. thermophilum**, *S. abbasi*
X. innexi	Lengyel et al. 2005	*S. scapterisci*
X. ishibashii	Kuwata et al. 2013	*S. aciari*
X. japonica	Nishimura et al. 1994	*S. kushidai*
X. khoisanae	Ferreira et al. 2013	*S. khoisanae, S. jeffreyense, S. saccharii*
X. koppenhoeferi	Tailliez et al. 2006	*S. scarabaei*
X. kozodoii	Tailliez et al. 2006	*S. arenarium*
X. magdalenensis	Tailliez et al. 2012	*S. australe*
X. mauleonii	Tailliez et al. 2006	undescribed
X. miraniensis	Tailliez et al. 2006	undescribed
X. nematophila	Poinar and Thomas 1965	*S. carpocapsae*
X. poinarii	Akhurst, 1983	*S. glaseri, S. cubanum*
X. romanii	Tailliez et al., 2006,	*S. puertoricense*
X. stockiae	Tailliez et al. 2006	*S. siamkayai*
X. szentirmaii	Lengyel et al. 2005	*S. rarum*
X. thuongxuanensis	Kämpfer et al. 2017	*S. sangi*
X. vietnamensis	Tailliez et al. 2010	*S. eapokense*

* *nomina nuda*

Table S2. *Xenorhabdus* strains included in this study, their hosts, and GeneBank accession numbers for analysed genes

nematodes		bacteria					
isolate	origin / GPS	strain	GenBank accession no.				
			16S rDNA	*rec*A	*gyr*B	*dna*N	*glt*X
S. poinari S11/041	51°23'23"N 22°31'34"E	Xb041	MG995578	MH0011599	MH0011601	MH0011594	MH0011604
S. poinari S11/057	50°46'38"N 22°02'28"E	Xb057	MG995577	MH0011598	MH0011602	MH0011595	MH0011603
S. poinari S11/139	50°39'47"N 23°03'09"E	Xb139	MG995576	MH0011597	MH0011600	MH0011596	MH0011605

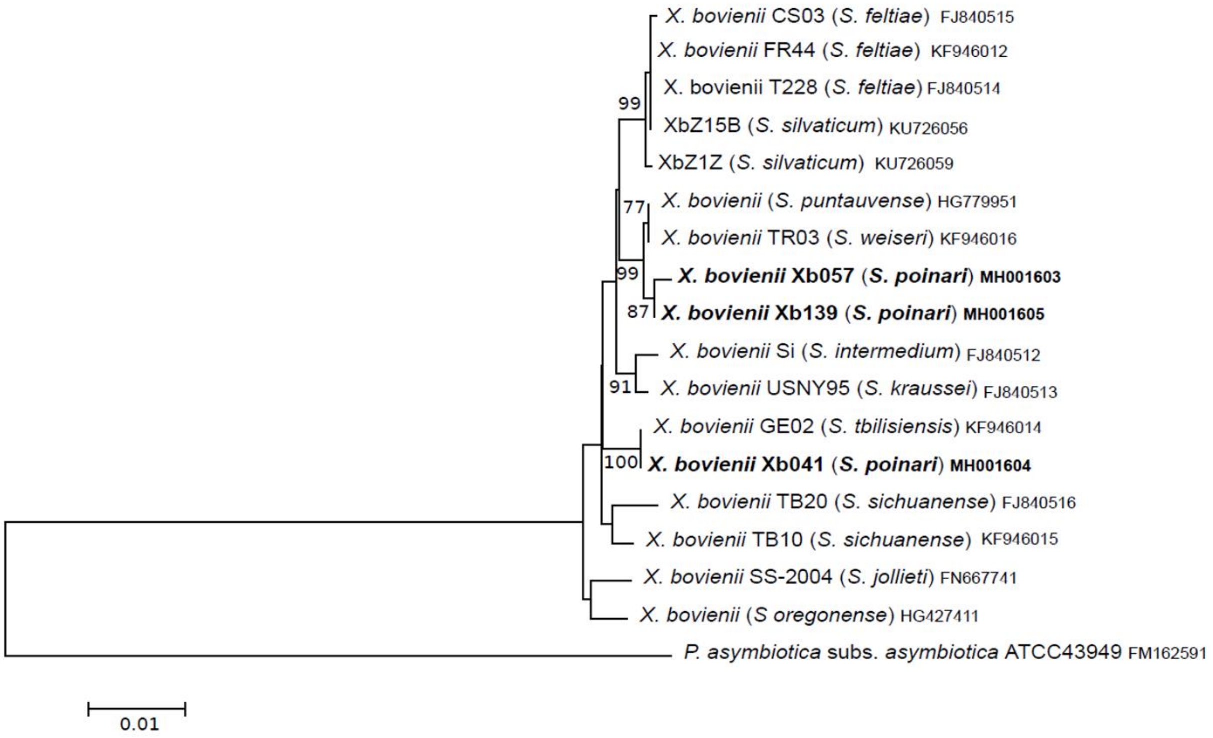

Fig. S1. Neighbor-joining tree showing the phylogenetic relationships of *S. poinari* microsymbionts studied (bolded) with *X. bovienii* strains based on *gltX* gene sequences. Bootstrap values based on 1000 replicates >70% are indicated at the branching points. The scale bar presents the number of nucleotide substitutions per site. The sequence of *Photorhabdus asymbiotica* subsp. *asymbiotica* was used as an outgroup

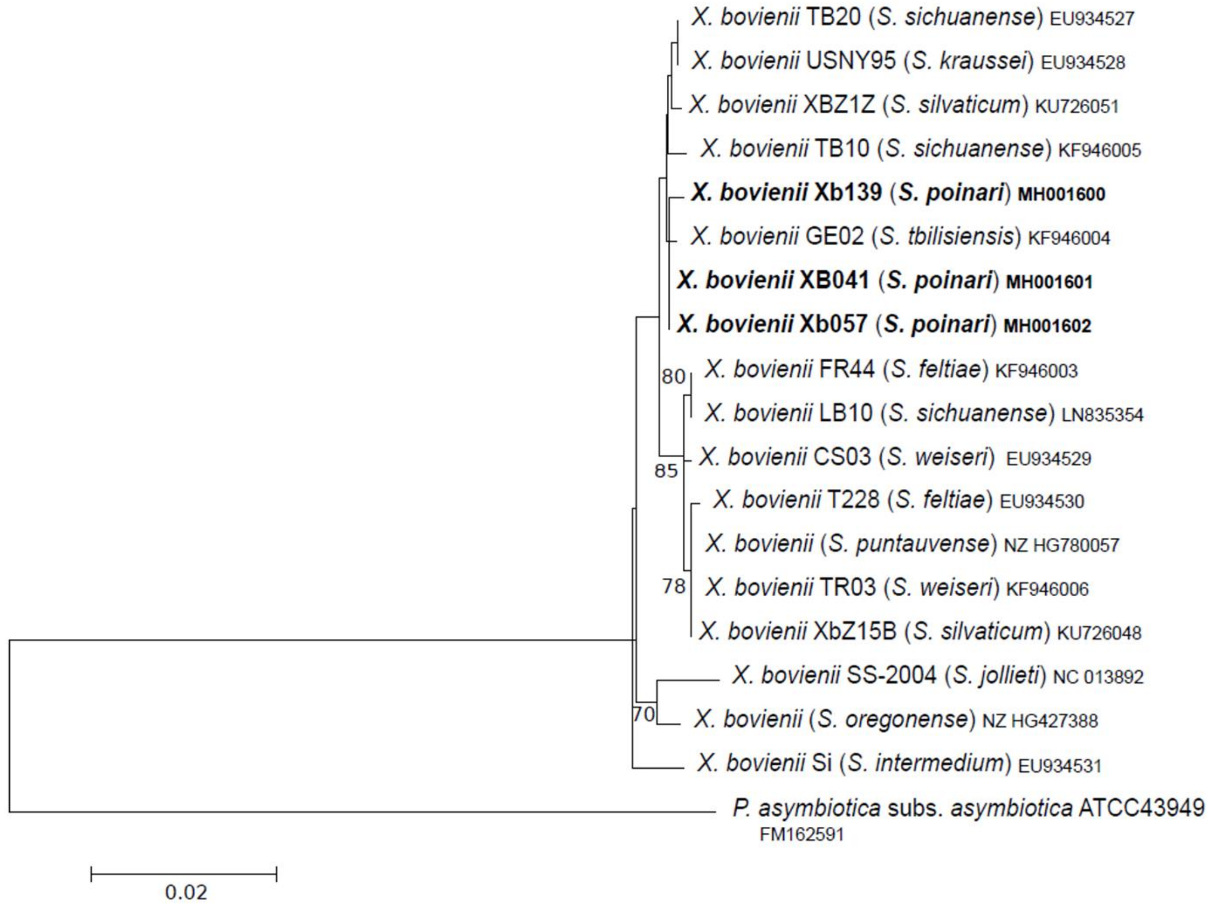

Fig. S2. Neighbor-joining tree showing the phylogenetic relationships of *S. poinari* microsymbionts studied (bolded) with *X. bovienii* strains based on *gyrB* gene sequences. Bootstrap values based on 1000 replicates >70% are indicated at the branching points. The scale bar presents the number of nucleotide substitutions per site. The sequence of *Photorhabdus asymbiotica* subsp. *asymbiotica* was used as an outgroup

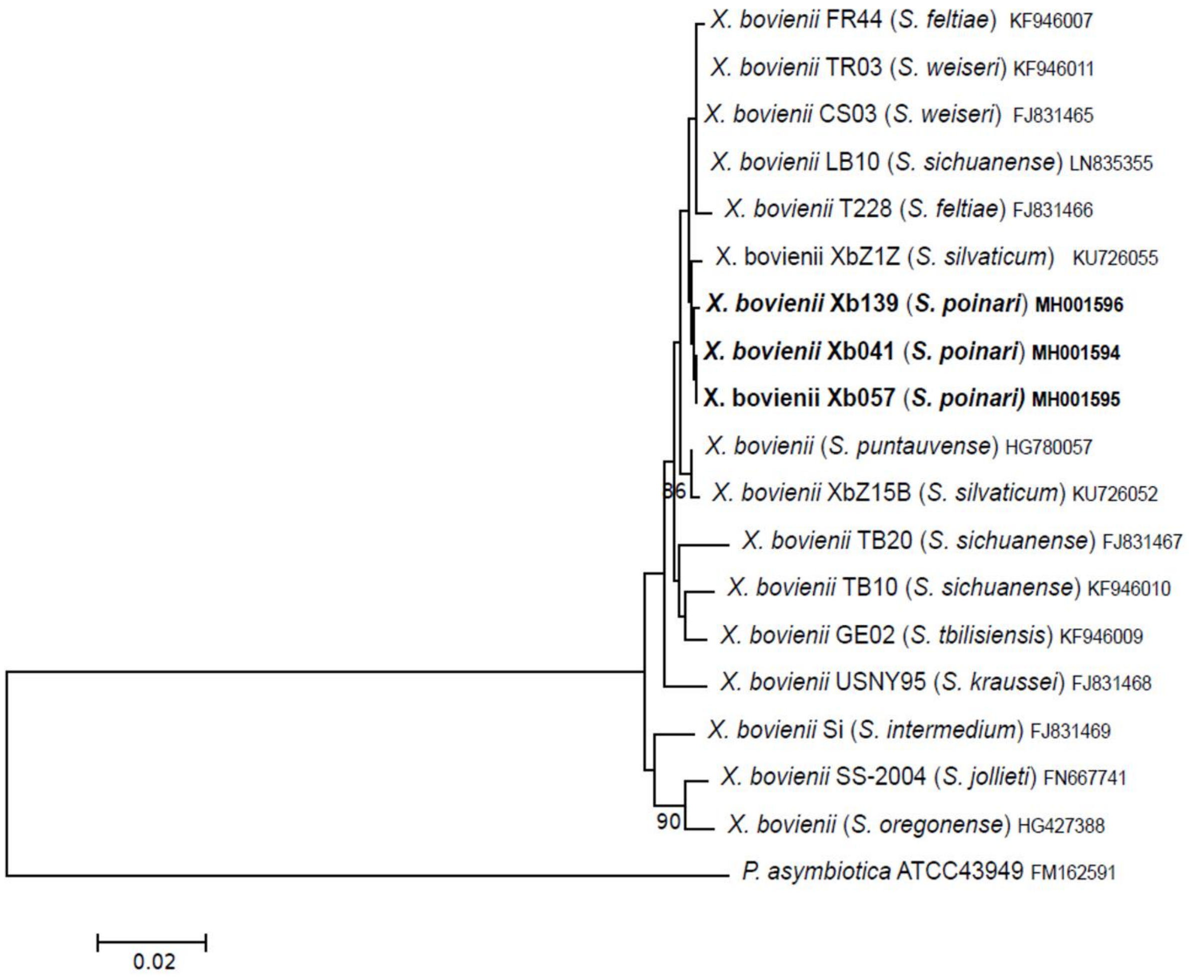

Fig. S3. Neighbor-joining tree showing the phylogenetic relationships of *S. poinari* microsymbionts studied (bolded) with *X. bovienii* strains based on *dnaN* gene sequences. Bootstrap values based on 1000 replicates >70% are indicated at the branching points. The scale bar presents the number of nucleotide substitutions per site. The sequence of *Photorhabdus asymbiotica* subsp. *asymbiotica* was used as an outgroup

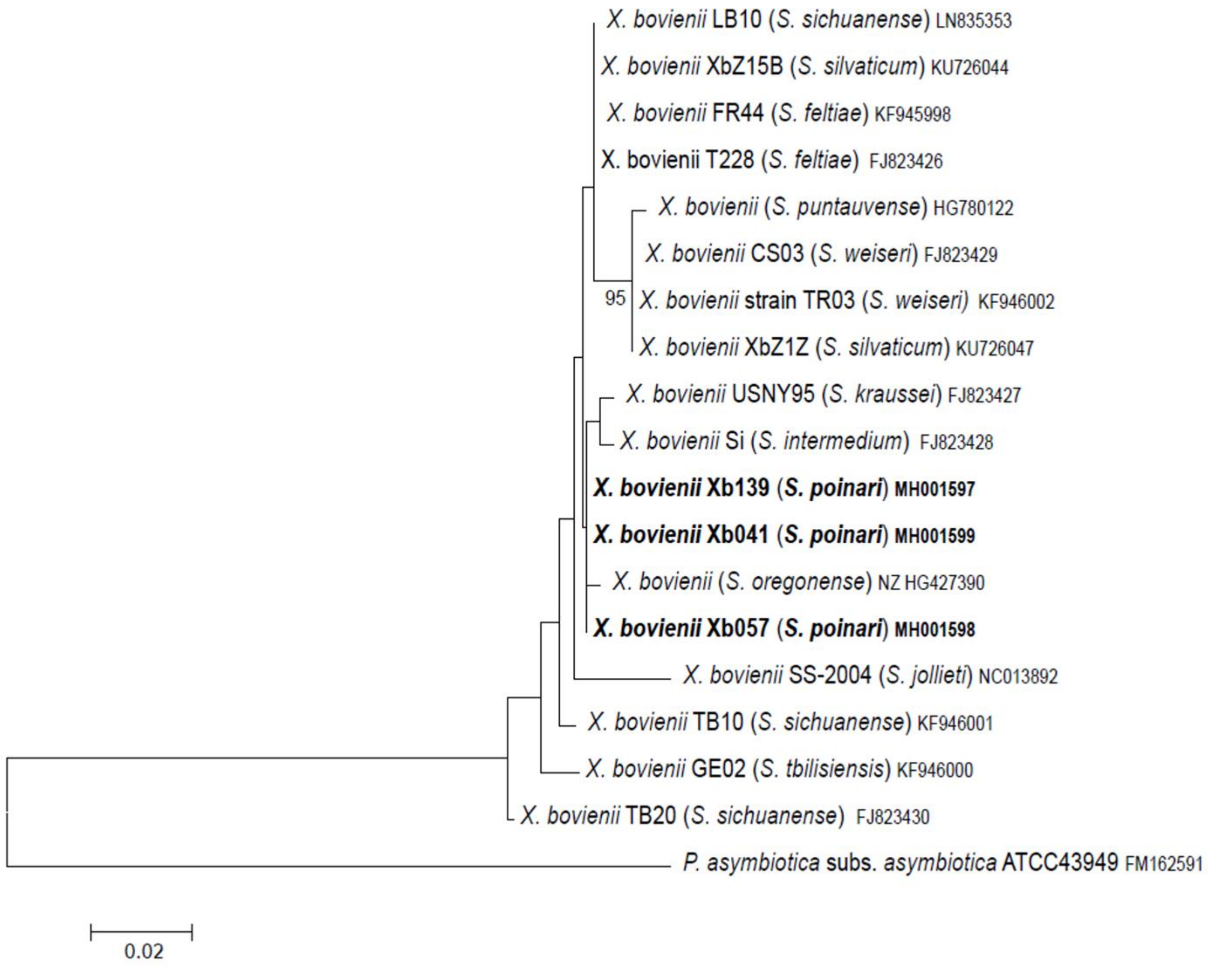

Fig. S4. Neighbor-joining tree showing the phylogenetic relationships of *S. poinari* microsymbionts studied (bolded) with *X. bovienii* strains based on *recA* gene sequences. Bootstrap values based on 1000 replicates >70% are indicated at the branching points. The scale bar presents the number of nucleotide substitutions per site. The sequence of *Photorhabdus asymbiotica* subsp. *asymbiotica* was used as an outgroup

Supplemental references

Akhurst RJ (1983) Taxonomic study of *Xenorhabdus*, a genus of bacteria symbiotically associated with insect pathogenic nematodes. Int J Syst Bacteriol 33(1):38-45. doi: 10.1099/00207713-33-1-38

Akhurst RJ, Boemare NE (1988) A numerical taxonomic study of the genus *Xenorhabdus* (Enterobacteriaceae*)* and proposed elevation of the subspecies of *X. nematophilus* to species. J Gen Microbiol 134(7):1835–1845. doi: 10.1099/00221287-134-7-1835

Dreyer J, Malan AP, Dicks LMT (2017) Three novel *Xenorhabdus-Steinernema* associations and evidence of strains of *X. khoisanae* switching between different clades. Curr Microbiol 74(8):938–942. doi: 10.1007/s00284-017-1266-2

Ferreira T, van Reenen CA, Endo A, Sproer C, Malan AP, Dicks LMT (2013) Description of *Xenorhabdus khoisanae* sp. nov., the symbiont of the entomopathogenic nematode *Steinernema khoisanae*. Int J Syst Evol Micr 63(9):3220–3224. doi: 10.1099/ijs.0.049049-0

Kämpfer P, Tobias NJ, Ke LP, Bode HB, Glaeser SP (2017) *Xenorhabdus thuongxuanensis* sp. nov. and *Xenorhabdus eapokensis* sp. nov., isolated from *Steinernema* species. Int J Syst Evol Microbiol 67(5):1107-1114. doi: 10.1099/ijsem.0.001770

Kuwata R, Qiu L-H, Wang W, Harada Y, Yoshida M, Kondo E, Yoshiga T (2013) *Xenorhabdus ishibashii* sp. nov., isolated from the entomopathogenic nematode *Steinernema aciari*. Int J Syst Evol Microbiol 63(5):1690–1695. doi: 10.1099/ijs.0.041145-0

Lengyel K, Lang E, Fodor A, Szallas E., Schumann P., Stackebrandt E (2005) Description of four novel species of *Xenorhabdus*, family *Enterobacteriaceae*: *Xenorhabdus budapestensis* sp. nov., *Xenorhabdus ehlersii* sp. nov., *Xenorhabdus innexi* sp. nov., and *Xenorhabdus szentirmaii* sp. nov. Syst Appl Microbiol 28(2):115–122. doi: 10.1016/j.syapm.2004.10.004

Nishimura Y, Hagiwara A, Suzuki T, Yamanaka S (1994) *Xenorhabdus japonicus* sp. nov. associated with the nematode *Steinernema kushidai.* World J Microb Biot 10(2):207–210. doi: 10.1007/BF00360889

Poinar GO, Thomas GM (1965) A new bacterium, *Achromobacter nemathopilus* sp. nov. (*Achromobacteriaceae: Eubacteriales*), associated with a nematode. Int B Bact Nomencl T 15:249-252

Somvanshi VS, Lang E, Ganguly S, Swiderski J, Saxena AK, Stackebrandt E (2006) A novel species of *Xenorhabdus*, family *Enterobacteriaceae*: *Xenorhabdus indica* sp. nov., symbiotically associated with entomopathogenic nematode *Steinernema thermophilum* Ganguly and Singh, 2000. Syst Appl Microbiol 29(7):519–525. doi: 10.1016/j.syapm.2006.01.004

Tailliez P, Laroui C, Ginibre N, Paule A, Pages S. Boemare N (2010) Phylogeny of *Photorhabdus* and *Xenorhabdus* based on universally conserved protein-coding sequences and implications for the taxonomy of these two genera. Proposal of new taxa. *X. vietnamensis* sp. nov., *P. luminescens* subsp. *caribbeanensis* subsp. nov., *P. luminescens* subsp*. hainanensis* subsp. nov., *P. temperata* subsp. *khanii* subsp. nov., *P. temperata* subsp. *tasmaniensis* subsp. nov., and the reclassification of *P. luminescens* subsp. *thracensis* as *P. temperata* subsp*. thracensis* comb. nov. Int J Syst Evol Micr 60(8):1921–1937. doi: 10.1099/ijs.0.014308-0

Tailliez P, Pages S, Edgington S, Tymo LM, Buddie AG (2012) Description of *Xenorhabdus magdalenensis* sp. nov., the symbiotic bacterium associated with *Steinernema australe*. Int J Syst Evol Micr 62(8):1761–1765. doi: 10.1099/ijs.0.034322-0

Tailliez P, Pages S, Ginibre N, Boemare N (2006) New insight into diversity in the genus *Xenorhabdus*, including the description of ten novel species Int J Syst Evol Micr 56(12):2805–2818. doi: 10.1099/ijs.0.64287-0
